# 

**Published:** 1861-03

**Authors:** 


Vol. II.] MARCH, 1861. [No. 3.

### THE CHICAGO

### MEDICAL EXAMINER

A Monthly Journal, devoted to the Educational, Scientific, and practical interests of the Medical Profession.

EDITED BY

N. S. DAVIS, M. D.

Professor of Principles and Practice of Medicine r nd Clinical Medicine in the Medical Department of Lind University

Terms—$2.00 per annum, in advance.

CHICAGO:

Wm. Cravens & Co. Printers and Publishers,

No. 132 LAKE STREET.

